# Allometric scaling of decompression sickness risk in terrestrial mammals; cardiac output explains risk of decompression sickness

**DOI:** 10.1038/srep40918

**Published:** 2017-02-02

**Authors:** Andreas Fahlman

**Affiliations:** 1 Fundación Oceanogràfic de la Comunitat Valenciana, c/Gran Vía Marqués del Turia 19 46005, Valencia España.

## Abstract

A probabilistic model was used to predict decompression sickness (DCS) outcome in pig (70 and 20 kg), hamster (100 g), rat (220 g) and mouse (20 g) following air saturation dives. The data set included 179 pig, 200 hamster, 360 rat, and 224 mouse exposures to saturation pressures ranging from 1.9–15.2 ATA and with varying decompression rates (0.9–156 ATA • min^−1^). Single exponential kinetics described the tissue partial pressures (P_tiss_) of N_2_: P_tiss_ =  ∫(P_amb_ – P_tiss_) • τ^−1^ dt, where P_amb_ is ambient N_2_ pressure and τ is a time constant. The probability of DCS [P(DCS)] was predicted from the risk function: P(DCS) = 1−e^−*r*^, where *r* = ∫(P_tiss_N_2_ − Thr − P_amb_) • P_amb_^–1^ dt, and Thr is a threshold parameter. An equation that scaled τ with body mass included a constant (c) and an allometric scaling parameter (*n*), and the best model included *n*, Thr, and two c. The final model provided accurate predictions for 58 out of 61 dive profiles for pig, hamster, rat, and mouse. Thus, body mass helped improve the prediction of DCS risk in four mammalian species over a body mass range covering 3 orders of magnitude.

Decompression sickness (DCS) is a potential hazard for divers and aviators that is difficult to study in humans without exposing them to potential harm. Despite over 100 years of research, the etiology of DCS is still poorly understood. For example, there appears to be considerable within- and between-subject variability in susceptibility and even when developed guidelines are scrupulously followed, DCS still occurs in some divers. It is universally accepted that the bubbles formed during the decompression phase from the elevated blood and tissue gas tension may cause direct problems such as vascular emboli, while the large variation in outcome may indicate secondary issues such as involvement of the immune system^1^ and modification of the endothelial surface of blood vessels^2^,^3^. While previous work has shown that the probability of DCS [P(DCS)] is seldom a certainty of zero for any hyperbaric exposure^4^, data suggest that P(DCS) correlates with pressure, duration of pressure exposure, and ascent rate^4^,^5^,^6^,^7^.

The theoretical basis for this correlation can be found when investigating how inert gases are taken up and removed. It is assumed that tissue inert gas tension (P_tiss_) is predicted by:


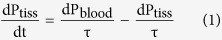


where P_blood_ is the arterial blood tension of the inert gas (ATA) and τ is the tissue time constant (min). The solubility of N_2_ in plasma and blood is low^8^; its removal and uptake are therefore assumed to be perfusion-limited^9^, and P_blood_ equal to the ambient partial pressure of that gas, i.e. assuming instant equilibrium at the lung-blood barrier. τ determines the tissue uptake and removal rate, and is a physiologically relevant parameter related to the size (volume, V) of the animal, the solubility of the gas in the blood and tissue (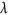), and the cardiac output (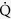, L min^−1^) as:


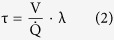


The average τ for the whole animal is a composite of all the different tissue compartments with differing gas solubilities and is determined by 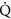. Consequently, variation in 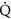 causes variation in uptake and removal rates of the inert gas and may therefore alter P(DCS).

A number of physiological variables such as body temperature, body mass (*M*_b_), exercise, sex, adiposity, age, Doppler bubble grades, and patent foramen ovale have been suggested to alter P(DCS)^10^,^11^,^12^,^13^,^14^, but among these only *M*_b_ has been shown to correlate with P(DCS) within and between species^10^,^15^,^16^. As 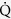 scales allometrically with *M*_b_^17^, DCS risk should also scale with *M*_b_ unless there are differences in susceptibility between species.

In the current study it was hypothesized that DCS risk between species of varying size can be explained by differences in *M*_b_ and 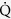. If so, findings from studies using animals of different body size or species could be compared. If, on the other hand, susceptibility in DCS risk is strongly influenced by physiological properties that are mass independent and species-specific, then mass-based extrapolation would not be straightforward in predicting DCS risk between species.

## Results

### Model iterations

Initially, the model was fitted to estimate a separate τ for each species, i.e. each species was fitted to model M3. As data for pigs were available for two separate body mass ranges (20 and 70 kg), two separate τ values were estimated for this species. The resulting τ for each species was plotted against *M*_b_, and the allometric equation (Eq. 8, see Materials and Methods) used to determine c (2.07 ± 0.36) and *n* (0.56 ± 0.04, r^2^ = 0.99, P < 0.01). In Eq. 8, c is a constant that together with *n*, the allometric mass-exponent, helps scale τ for variation in *M*_b_.

The results for 8 nested models are summarized in Table 1. Of these, the most parsimonious model included two values for c, one *n*, and a supersaturation threshold parameter (Thr, M8). When *n* was included in the model it reduced c by between 52–98% (M1–58.5 min • kg^−1^ vs. M3–1.29 min • kg^−1^ and M5 vs M7). When Thr was included in models that contained *n*, the parameter estimate for both c and *n* increased (M3 vs M4 and M7 vs M8).

In additional models Thr, and *n* were adjusted for each species to test if there were differences in susceptibility attributable to individual species. None of these models warranted inclusion of separate parameters for each species. A single value for *n* accounted for the variation between species.

### Goodness of Fit

The goodness of fit test for the best model was used to determine if systematic changes in the model predictions occurred with changes in depth and decompression rates within and between species (Table 2). In summary, the model predictions of the dive profiles most often failed either at the highest or lowest depth or decompression rates. Despite an improved fit, the addition of Thr or *n* had minimal impact on the failure rate for the data (e.g. M1 v.s M2 or M3, or M5 vs M6 and M7). Inclusion of both Thr and *n* improved the goodness of fit for all species as compared to models with only *c* (M1 vs M4 and M5 vs M8).

For the best model (M8, Tables 1 and 2), one dive profile for the rat (depth 6.3 ATA, decompression rate 43 ATA • min^−1^) under predicted [P(DCS)_est_ = 0.49] the observed DCS rate [P(DCS)_obs_ = 0.87, Table 2]. However, the observed number of animals with DCS for this profile was unusual as it was higher than two other profiles with a more severe decompression rate [P(DCS)_obs_ =0.67 for 54 ATA • min^−1^ and P(DCS)_obs_ = 0.73 for 64 ATA • min^−1^]. Model M8 also failed to accurately predict the P(DCS) for two pig dive profiles. One dive profile for the 20 kg pig (depth 3.4 ATA) over predicted P(DCS)obs [P(DCS)_est_ = 0.40 vs. P(DCS)_obs_ = 0.00] while the profile with the highest pressure for the 70 kg (depth 2.8 ATA) under predicted the observed DCS rate [P(DCS)_est_ = 0.31 vs. P(DCS)_obs_ = 0.80, Table 2].

## Discussion

The current study investigated the assumption that DCS risk is governed by inert gas dynamics and can be allometrically scaled between species. The results suggest that the probability of DCS for air saturation dives in mammals can be scaled based on the variation in 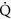 ranging in *M*_b_ over 3 orders of magnitude. The allometric scaling constant estimated from these data is within the range of expected values based on the hypothesis that risk is directly correlated with 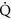. This suggests that among the species investigated, 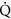 explains most of the variation in P(DCS). These results may help improve our understanding of the underlying mechanism of the etiology of DCS.

Mathematical models are useful to explore complex physiological systems, but often have numerous assumptions and limitations. The current study relies on a theoretical model that describes how N_2_ is taken up and removed from tissues during a hyperbaric exposure. The model assumes that the probability to experience severe DCS symptoms is related to the integrated supersaturation, or risk. As such, the model cannot discriminate between symptoms caused by bubbles formed in the tissues or circulatory system. In addition, the current effort uses historical data to investigate if *M*_b_ can be used to scale DCS between terrestrial mammals of different size, and hyperbaric profiles. These data are limited in scope and detailed information is not available for certain variables. For example, the individual weight for each animal was not available for many of the studies used and the mean value was used. Therefore, the model did not evaluate within-species differences, but looked at differences between species. The current analysis extreme compression extreme compression and decompression profiles that are not used in human dive operations. In addition, a majority of the observations used in this modeling effort comes from animals weighing less than1 kg. Consequently, the results and conclusions presented here should be interpreted carefully.

Despite data from a variety of terrestrial mammal species in DCS research, it is still unknown if differences in susceptibility are caused merely by variation in *M*_b_^7^,^10^,^15^ or if additional factors play a role^1^,^18^,^19^,^20^. Therefore it is difficult to compare results from different laboratories that use different animal models. While it is known that variation in *M*_b_ and decompression rate affect the probability of DCS for a given dive profile^10^,^16^, limited information exists to scale risk between species and for different dive profiles. Analysis of historical data supports the idea that DCS risk from differing dive profiles correlates with M_b_^7^,^10^. However, it is not known whether this variation in susceptibility within and between species is caused by factors other than changes in 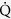.

Animal studies have demonstrated involvement of the immune system^1^, and increased levels of NO appear to modify the endothelial surface of blood vessels and reduce bubble formation^2^. Additionally, there are reports suggesting that blood microparticle (MP) levels increase with increasing decompression stress in both terrestrial and marine mammals^3^,^22^. Thus, variation in immune function may alter susceptibility among different animal species. For example, acclimation and immune competence have been shown to alter DCS risk in a number of animal models^1^,^18^,^23^,^24^, and some studies indicate that repeated exposures reduce DCS risk in humans^25^,^26^,^27^. Consequently, variation in immune function, vascular morphology, or acclimation through repeated exposures may alter susceptibility.

Alternatively, differences in susceptibility could be caused by variation in gas uptake and removal, and/or bubble formation and growth. Severe decompression rates may cause different levels of bubble formation, which itself affect blood flow and gas exchange. For example, the physical process of bubble formation and gas dynamics operate on different time scales. Bubble formation may require a certain level of supersaturation for bubbles to seed and grow, while inert gas removal depends on the perfusion rate. The much higher mass-specific 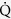 for small animals increases the uptake and removal rate of gases, thus shortening the period during which small animals experience supersaturation. This shorter period of supersaturation in smaller animals may require higher levels of supersaturation for bubbles to form and grow. In such a scenario, τ and Thr would vary between species in complex ways that cannot be predicted through allometric changes in 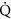. Evidence for differences in P(DCS) between species other than those from changes in *M*_b_ comes from unpublished data in guinea pigs weighing 500–600 g where the DCS risk was equal to or lower as compared to rats weighing 200–300 g (Lillo, personal communication). The current study was therefore conducted to test the hypothesis that variation in P(DCS) between species can be explained by allometric changes in 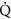.

While previous descriptive dose-response models have suggested that DCS risk can be scaled between species^5^,^7^,^15^, those models did not account for variation in the decompression rate between species, even though this variable is known to affect DCS risk, and to vary with *M*_b_. Consequently, an alternative probabilistic model that accounts for variation in ascent rate was used in the current study. Probabilistic models for DCS have been used successfully to model the P(DCS) for human, pig and sheep data for a number of compression and decompression profiles and different diluent dive gases^4^,^6^,^21^,^28^,^29^,^30^. Probabilistic models offer an advantage over previous models as they account for accumulation of risk during the ascent (decompression). The model used in the current study was modified to include variation in risk with *M*_b_ (Eq. 8), so that variation in P_tiss_ (saturation depth) and ascent rate were also accounted for by accumulating risk during the decompression. This allows us to test if τ, which relates blood flow and animal size (Eqs 7 and 8) to P(DCS), is a physiologically important parameter.

The tissue time constant provides information about the removal rate of inert gas. While this parameter is a theoretical construct^6^,^31^, it offers information about the physiological basis of DCS, and allows general predictions how risk may scale between species. First, due to the low solubility of N_2_ in tissues and blood, it is generally assumed that N_2_ removal is perfusion-limited^9^. If inert gas exchange is governed by 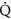, τ should increase allometrically similar to the relationship between 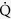 and *M*_b_ (Eq. 7) 32. The allometric mass-exponent was 0.37 ± 0.04 (model M8, Table 1), which is within the expected range of values of 0.12–0.33^17^,^33^,^34^,^35^ if variation in P(DCS) can be explained by differences in 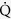. This finding provides preliminary evidence that the underlying mechanism of DCS is a physical process where incomplete removal of inert gas result in supersaturation and gas bubble formation. These results provide an alternative explanation for changes in P(DCS) following exercise, nitric oxide synthase (NOS) inhibition, or immune challenge^1^,^2^,^18^,^20^,^36^, as variation in 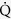 may also explain the result in those studies. Consequently, studies should consider assessing 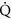 to assure that there are no confounding effects.

**Figure 1:**
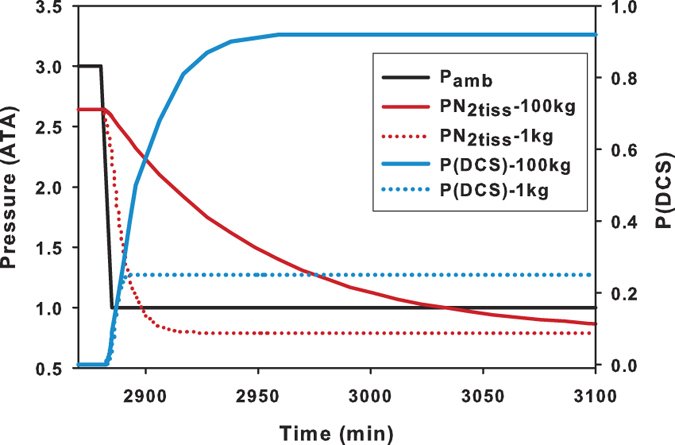
Tissue gas tension (P_tiss_) and probability of decompression sickness (P[DCS]) for animals of differing body masses (1 and 100 kg) for an air saturation dive to 3 ATA (20 m) with a bottom duration of 2880 min and a decompression rate of 0.4 ATA • min^−1^ for the slowest time constant (c1 for model M8, Table 1; τ_1kg_ = 6.2 min, τ_100 kg_ = 31.3 min, Eq. 8).

Probabilistic models may provide a method to predict the relative risk of DCS for dives of varying hyperbaric profiles and allow scaling between species. This could significantly enhance the ability to interpret results between species and allow testing high-risk dives or new therapeutics in small animal models for prediction of DCS risk for human dives. The model presented in the current study allows us to assess how changes in 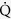 alter risk. For example, using the results for model M8, the two values of τ for a 20 g mouse are 0.3 sec and 1.6 min (Table 1 and Eq. 8), while for a 20 kg pig the same τ values are 2.9 sec and 18 min, respectively. Thus, the variation in τ with *M*_*b*_ suggests that the inert gas is more rapidly removed from a smaller animal, and P(DCS) should therefore be less for a given dive profile. In addition, this model would allow testing how changes in 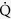 during exercise, stress, or ingestion of compounds that alter blood flow (e.g. coffee), might affect P(DCS). Figure. 1 illustrates the model predictions of P(DCS) for an air saturation dive profile to 3 ATA for a 1 kg versus a 100 kg mammal. In this model, both the large and small animals are saturated at the start of decompression. In the smaller animal, P_tiss_ is rapidly reduced as N_2_ is removed, and when reduced below the threshold (numerator = 0 in Eq. 6) the risk stops accumulating (Fig. 1). The greater τ in larger animals allows the risk to accumulate for longer, resulting in a higher P(DCS).

The current analysis is limited to extreme air dive profiles, in which the animal is held at depth until saturation is assumed and then decompressed at a rate that causes a high incidence of severe and easily observable DCS symptoms. For the data used, the level of movement differed between studies and/or species. For example, the rats were placed in a cage that rotated which helped make sure that they walked at constant speed throughout the hyperbaric exposure and post-decompression period. In the pig, on the other hand, movement was voluntary and varied between individual animals. However, the model results did not warrant inclusion of parameters that varied susceptibility between species, other than through *M*_b._ This suggests that any variation in experimental design or sensitivity towards gas emboli did not exist or was minimal as compared to variation in 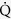. In addition, the value of *n* was close to that predicted from allometric scaling of 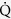 (model M8, Table 1), which agrees with the hypothesis that most variation in P(DCS) is related to variation in blood flow.

The results presented in the current study indicate that *M*_b_ can be successfully used to scale P(DCS) between species. The risk of DCS scales with *M*_b_ with a mass-exponent that suggest that most of the variation in P(DCS) is related to variation in 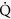. Thus, scaling P(DCS) between mammalian species could be a powerful way to test high risk hyperbaric compression/decompression profiles or candidate drugs for therapeutic purposes in small animal models.

## Materials and Methods

### Data set and case descriptions

The data used in this study were taken from previously published animal experiments and have been reported in detail elsewhere (Table 3). A brief summary of each species and the corresponding experimental procedure is described below. Only severe DCS symptoms were considered (respiratory distress, hind limb paralysis, death) to avoid the inherent variation in scoring symptoms between different individuals and studies. Thus, signs of DCS were in most cases severe enough to leave no diagnostic ambiguity. In addition, only single exposure air saturation dive profiles were included to avoid confounding results from differences between gas mixtures or repeated exposures^21^,^23^.

#### (i) Pig

Pigs (*Sus scrofa*, 17–23 kg, Table 3): The data set contained 125 previously published, well-documented hyperbaric exposures from juvenile male Yorkshire pigs^37^, an additional 41 hyperbaric exposures^38^,^39^ of male Yorkshire pigs (same vendor) of larger size (59–79 kg) were also included. The hyperbaric experiments of the larger pigs were performed in a similar chamber, and scored by the same research group as for the smaller pigs. The time at depth, compression and decompression rates were similar between the large and small pigs (Table 3).

#### (ii) Hamster

Data were taken from a study that included both variable times at depth and constant depth data, but only hyperbaric exposures of 30 min were used in our analysis^40^. The *M*_b_ of each individual hamster was not specified and an average *M*_b_ of 100 g was therefore assumed for each animal.

#### (iii) Rat

The rat data sets were collected from three separate studies^21^,^41^,^42^. The *M*_b_ of each rat was not reported; the average for each profile and study were used (Table 3). Each rat was exercised throughout the hyperbaric experiment and during the 15–30 min post-dive observation period by rotating the cylindrical cage (3 m • min^−1^) in which they were housed. From the three studies, only air saturation exposures were included.

#### (iv) Mouse

Dive exposures exceeding 15 min were included from three separate studies^5^,^7^,^43^. The mice were exercised in a rotating cage at 5 revolutions per min (rpm)^5^,^7^,^43^. As the diameter of the cage was not specified, the actual speed was unknown. The body mass of each mouse was not reported, and the average body mass of each group was therefore used (Table 3).

### Data Preparation

There was significant variation in the numbers of observations for the different species, e.g. 592 for mouse, 732 for hamster, 625 for rat, and 170 for pig (Table 3). Thus, most hyperbaric exposures came from animals that weighed <300 g. The numbers of observations for mouse, hamster and rat were therefore truncated while keeping the DCS rate the same for each profile. For example, if a dive profile had 30 observations with 18 DCS cases, the number of observations was reduced to 10 cases with 6 DCS cases. This reduced the data set from 2128 to 963 separate dive profiles, by reducing the number of observations for mouse (592 to 224), rat (625 to 360), and hamster (732 to 200), but keeping the P(DCS) the same for each separate dive profile. Smaller animals had systematically deeper dives of shorter duration and faster ascent rates (Table 3). Thus, by balancing the data set, model error was spread more evenly over the different depths and compression/decompression profiles, and minimized the chance that one species or dive profile might significantly influence the results.

### Hyperbaric exposure

The experiments include a variety of pressurization and depressurization sequences (Table 3). All hyperbaric exposures were performed in dry chambers of varying internal volumes.

Time 0 (T0) of a hyperbaric exposure marks the start of decompression. The time at which the animal was definitely free of DCS symptoms was defined as “T1”. This can be determined in many ways, but due to the inherent subjective aspects of scoring DCS symptoms in animals, T1 was set at the start of decompression. To accommodate the different experimental designs, “T2” was defined as the end of the observation period. Upon returning to 1 ATA the observation times ranged from 5 min for mice^5^ to 120 min for large pigs^38^,^39^.

### DCS risk assessment modeling

The probability of DCS [P(DCS)] was determined using maximum likelihood to search for the best-fitting parameters^6^. The probability of displaying DCS symptoms at a time T after a hyperbaric exposure is defined as:


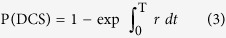


The probability of animals not displaying symptoms until time T is defined as:


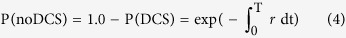


where *r* is the instantaneous risk^4^,^30^. The value of *r* was constrained to be > 0 whenever P_tiss_ > P_amb_, and r was forced to 0 at any time

P_tiss_ ≤ P_amb_, where P_amb_ is the ambient N_2_ pressure^4^.

The probability of developing DCS for a particular hyperbaric exposure is defined as a product of the probability of no DCS being observed over a given interval and the probability of DCS occurring from a time T1 until the end of the post decompression observation period (T2)^4^,^30^. Thus, P(DCS) was computed as:


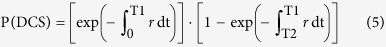


For the current study, T1 was at the start of the decompression. This approach is conservative and may result in some loss of temporal information, but reduces ambiguity in scoring DCS between studies. As the time when symptoms occurred was only detailed for the pigs, T2 was set at the end of the post decompression observation period.

### Model

Different definitions of *r* allow for different mechanisms of DCS to be tested^4^,^6^,^30^. In the current study, we defined r as the relative difference between P_tiss_ and P_amb_ above a threshold pressure (Thr, ATA)^4^,^6^,^30^,


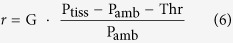


where G (gain) is a scaling factor (min^−1^) to be determined from the fitting procedure. Inclusion of Thr has been controversial^6^,^29^, but was tested in this study. As shown in Eq. 6, Thr is a level of tissue over-pressure (i.e., P_tiss_ > P_amb_) that can be sustained without incurring risk; only when (P_tiss_ − P_amb_) exceeds Thr is risk greater than zero. As in some previous DCS modeling efforts^6^,^44^, the contributions of O_2_, CO_2_, and water vapor to DCS risk were ignored.

### Tissue inert gas tension (P_tiss_)

We assumed single exponential gas kinetics to estimate P_tiss_ from a single or multiple compartments (Eq. 1). For multiple compartments, the model included additional values for τ, and inclusion was determined by the fitting procedure^4^.

### Effect of body mass on tissue time constants

The cardiac output varies with M_b_ as:


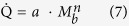


where a is a constant and *n* is an allometric mass-exponent. As the blood supply of O_2_ to the tissues is determined by the metabolic rate, the *n* for both metabolic rate and 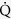 are similar. However, the variation in *n* varies considerably depending on the theory behind allometric scaling from 0.66–0.88^17^,^33^,^34^,^35^,^45^. In Eq. 2, V and λ (the volume of the animal and its overall N_2_ solubility) remain constant during a single dive, while 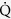 may vary and alter τ. Thus, τ was computed as:


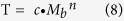


where c and *n* were fitted from the data. As τ is inversely related to 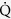 (Eq. 2), *n* in Eq. 8 should be 1-*n* for 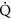, or approximately 0.12–0.33. Consequently, if uptake and removal are perfusion-limited and thus governed by cardiac output, this allows some basic predictions:

1. τ should correlate with M_b_ and be shorter (faster gas kinetics) for smaller species,
2. if all variation in P(DCS) varies with 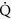, *n* should be 0.12–0.33, and
3. larger species should have greater P(DCS) for the same saturation dive profile.

### Model Analysis

The parameters were determined from the data by fitting the estimated P(DCS) to the actual outcome for each hyperbaric exposure, using Eqs 3 or 5. For models having more than one τ (calculated using Eq. 8 and c in models M5-M8, table 1), a second risk function was added with a separate c and G parameter, but sharing the Thr and *n*. The total risk for x number of tissues was therefore


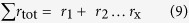


The method of maximum log-likelihood (LL) was used to search for best-fitting parameters, and the log-likelihood ratio (LRT) test was used to determine significance between nested models^6^. The null model, against which all other models were compared, contained no explanatory variables^6^. Several unique starting parameter-value sets were used to increase the likelihood of finding global rather than local LL maxima. Differences were considered significant at the P < 0.05 level.

### Goodness of Fit

To assess the goodness of fit for each model, the P(DCS) was computed for each dive profile and compared against the observed DCS. The binomial confidence limit of the observed DCS rate was computed, and if the P(DCS) for each model was outside the 95% confidence limit, the model estimate for that dive profile was assumed unsuccessful and given a 1. The unsuccessful models were summed, and the overall error for all profiles for each species was reported as a percent of the total number of profiles.

### Data availability

The model and data sets used in this study are freely available at the following link osf.io/w2x6z.

## Additional Information

**How to cite this article**: Fahlman, A. Allometric scaling of decompression sickness risk in terrestrial mammals; cardiac output explains risk of decompression sickness. *Sci. Rep.* **7**, 40918; doi: 10.1038/srep40918 (2017).

**Publisher's note:** Springer Nature remains neutral with regard to jurisdictional claims in published maps and institutional affiliations.

## Figures and Tables

**Table 1:** Log-likelihood (LL) estimates for each of the models.

Model	LL	c_1_	G_1_	Thr	*n*	c_2_	G_2_
Null	−656.5	—	0.86±0.04	—	—	—	—
M1	−618.4	58.5 ± 5.1	0.0094 ± 0.0004	—	0^*^	—	—
M2	−618.0	63 ± 7	0.010 ± 0.001	0.11 ± 0.13	0^*^	—	—
M3	−611.3	1.29 ± 0.22	0.25 ± 0.05	—	0.30 ± 0.02	—	—
M4	−607.1	2.74 ± 0.60	0.17 ± 0.03	0.41 ± 0.12	0.35 ± 0.02	—	—
M5	−607.1	85.6 ± 14.5	0.0065 ± 0.007	—	0^*^	0.053 ± 0.039	0.81 ± 0.0.45
M6	−603.5	98.8 ± 112.6	0.0092 ± 0.0012	0.37 ± 0.11	0^*^	0.040 ± 0.033	1.35 ± 0.99
M7	−599.1	4.86 ± 2.77	0.043 ± 0.028	—	0.31 ± 0.02	0.0085 ± 0.002	21.2 ± 6.3
M8	−589.8	6.25 ± 2.18	0.064 ± 0.023	0.52 ± 0.12	0.37 ± 0.04	0.017 ± 0.003	20.5 ± 5.8

Models included two gain parameters (G), a mass-exponent (*n*), and/or a threshold parameter (Thr) and two parameters that allowed the time constant (τ) to be calculated from Eq. 8 using a constant (c) and *n*. ^*^For models M1, M2, M5, and M6 *n* is set to 0, so τ does not vary between species.

**Table 2:** Goodness of fit for each of the models presented in Table 1.

Species	Null	M1	M2	M3	M4	M5	M6	M7	M8
Mouse	75	81	81	88	88	94	87	100	100
Hamster	20	60	60	40	80	40	60	60	100
Rat	68	73	77	77	77	77	68	82	95
Pig-20	64	93	93	86	93	93	93	86	93
Pig-70	25	75	75	50	75	75	75	75	75
All	62	79	80	77	83	82	79	85	95

The values shown are the percent successful dive profiles when comparing the observed and predicted DCS for each model and species. For example, a value of 81 indicates that Model M1 successfully predicted the observed P(DCS) outcome for 81% of Mouse profiles.

**Table 3:** Dive data sets used for modeling.

Species	Animals (N)	Profiles	DCS	PO_2_ (ATA)	*M*_b_ (kg)	Pressure range (ATA)	Ascent rate (ATM • min^−1^)	Time at depth (min)	Ref.
Mouse	120 (240)	6	73 (60%)	0.5−1.0	0.02	14.2−15.2	142−164	30	43
Mouse	64 (64)	8	26 (41%)	0.3	0.02	14.8	1.4−104	16	7
Mouse	40 (288)	2	16 (40%)	0.5	0.02	13.8−14.2	43	15	5
Hamster	200 (732)	5	110 (55%)	1.2−2.2	0.1	5.7−10.5	38−185	31	40
Rat	195 (195)	13	136 (70%)	1.0	0.26	6.26−7.26	2.2−89	60	42
Rat	45 (80)	3	32 (70%)	1.0	0.25−0.27	6.3	76	60−120	21
Rat	120 (350)	6	72 (60%)	1.1−1.6	0.23−0.25	6.3−7.7	75−96	120	41
Pig	138	14	70 (51%)	0.5−1.0	21.0 ± 0.0	2.5−5.6	0.9	1440	46
Pig	41	4	15 (37%)	0.4−0.6	69.2 ± 4.0	1.9−2.8	0.9	1440	38,39
**Total**	**963 (2128)**	**61**	**550**	**0.3−2.2**	**0.02−69.2**	**1.9−15.2**	**0.9−185**	**15−1440**	

Data include number of animals (Animals), number of distinct dive profiles (Profiles), the number of DCS cases (DCS, and percentage in parenthesis), the O_2_ partial pressure at depth (PO_2_), the average reported body mass (kg), the pressure range (total ambient pressure) for the different dive profiles, the ascent (decompression) rate, and the reference where the data was published. Values within parenthesis for number of animals are the total number of animals used in the study. The data were reduced to minimize the influence of the much larger sample size for mouse, rat and hamster (see Material and methods).
